# Early Detection of Transient Hypoparathyroidism After Total Thyroidectomy: A Single-Center Preliminary Study

**DOI:** 10.3390/medicina62061137

**Published:** 2026-06-10

**Authors:** Marco Marian, Mihai Rosu, Cristi Tarta, Amadeus Dobrescu, Dan Brebu, Ionut Flaviu Faur, Andrei Korodi, Ioana Adelina Faur, Stefania Bunceanu, Dana Stoian

**Affiliations:** 1 Doctoral School, “Victor Babes” University of Medicine and Pharmacy, Eftimie Murgu Square, No. 2, 300041 Timisoara, Romania; marian.marco@umft.ro (M.M.); adelina.clim@umft.ro (I.A.F.); stefania.bunceanu@umft.ro (S.B.); 2 Center of Molecular Research in Nephrology and Vascular Disease, Faculty of Medicine, “Victor Babes” University of Medicine and Pharmacy, Eftimie Murgu Square, No. 2, 300041 Timisoara, Romania; stoian.dana@umft.ro; 3 1st Surgery Clinic, Emergency Clinical County Hospital of Arad, Andreny Karoly Str., No. 2–4, 310037 Arad, Romania; rosu.mihai@uvvg.ro; 4 Researching Future Surgery II Research Center, Department X, Discipline of General Surgery II, Faculty of Medicine, “Victor Babes” University of Medicine and Pharmacy, Eftimie Murgu Square, No. 2, 300041 Timisoara, Romania; dobrescu.amadeus@umft.ro (A.D.); brebu.dan@umft.ro (D.B.); flaviu.faur@umft.ro (I.F.F.); 5 2nd Surgery Clinic, Emergency Clinical County Hospital of Arad, Andreny Karoly Str., No. 2–4, 310037 Arad, Romania; 6 Department of Medicine, Faculty of Medicine, “Vasile Goldis” Western University of Arad, Liviu Rebreanu Str., No. 86, 310048 Arad, Romania; 7 Department of Internal Medicine II, Discipline of Endocrinology, Faculty of Medicine, “Victor Babes” University of Medicine and Pharmacy, E. Murgu Square, No. 2, 300041 Timisoara, Romania

**Keywords:** transient hypoparathyroidism, thyroidectomy, hypocalcemia, parathyroid hormone, thyroid gland weight, postoperative complications, central neck dissection, risk factors, biomarkers, thyroid cancers

## Abstract

*Background and Objectives*: Post-thyroidectomy hypoparathyroidism (hypoPTH) is the most common complication of total thyroidectomy. Transient hypoPTH was defined as postoperative day 1 (POD1) intact parathyroid hormone (PTH) < 15 pg/mL and/or symptomatic hypocalcemia (<8.0 mg/dL), requiring supplementation, resolving within six months. We evaluated POD1 calcium, PTH, and their combination; identified preoperative predictors; and compared absolute with percent-change metrics. *Materials and Methods*: Participants comprised a retrospective single-center cohort of 380 consecutive adults undergoing total thyroidectomy between January 2023 and December 2025. Multivariable logistic regression identified preoperative predictors, and receiver operating characteristic (ROC) analysis evaluated POD1 biomarkers. Because both biomarkers are part of the outcome definition, a pre-specified sensitivity analysis re-evaluated POD1 PTH and ΔPTH against PTH-independent outcomes (POD1-calcium-defined hypocalcemia and permanent hypoPTH). Subgroups examined malignancy and central neck dissection (CND). *Results*: The cohort comprised 193 males (50.8%) and 187 females (49.2%), with a median age of 53 years (IQR 38–69). Indications were multinodular goiter (45.0%), differentiated thyroid cancer (37.9%), Graves’ disease (15.0%) and recurrent disease (2.1%). CND was performed in 9.5% of patients. Transient and permanent hypoPTH occurred in 132 (34.7%) and 11 (2.9%) patients. Thyroid gland weight was the sole independent preoperative predictor (OR 0.982, 95% CI 0.969–0.995, *p* = 0.008), with smaller glands conferring higher risk. Against the composite outcome, POD1 calcium and PTH yielded AUCs of 0.997 and 0.991 (combined 1.000), reflecting partial circularity. In the decoupled-outcome sensitivity analysis, POD1 PTH retained good-to-excellent discrimination for severe hypocalcemia (AUC 0.943) and permanent hypoPTH (AUC 0.976). Malignant cases showed a greater relative PTH decline than benign cases (−53.7% vs. −38.5%, *p* = 0.013) despite comparable absolute POD1 values, and CND did not increase risk. *Conclusions*: Combined POD1 calcium and PTH provided strong biochemical confirmation of transient hypoPTH, but the composite-outcome AUCs reflect internal definitional consistency rather than independent predictive performance; the decoupled-outcome AUCs (0.93–0.98) are the conservative benchmark. Thyroid gland weight was an inverse risk modifier with limited stand-alone utility. Multicenter prospective validation is required.

## 1. Introduction

Thyroid cancer is the most common endocrine malignancy worldwide, with 586,202 new cases reported by GLOBOCAN 2020 [1] and an age-standardized incidence that has continued to rise through 2021, particularly among women and younger adults [2,3]. Driven by this trend and by the widespread detection of small papillary carcinomas on neck ultrasound, total thyroidectomy is now among the most frequently performed endocrine operations [4]. The procedure is generally safe, but it carries a well-recognized risk of complications, of which post-thyroidectomy hypoparathyroidism (hypoPTH)—resulting from inadvertent devascularization, traumatic injury, or removal of the parathyroid glands—remains the most frequent and clinically significant [5].

The reported incidence of post-thyroidectomy hypoPTH varies substantially across the literature. A landmark systematic review and meta-analysis of 115 studies by Edafe et al. reported a median incidence of transient hypocalcemia of 27% (interquartile range [IQR] 19–38%) and permanent hypocalcemia of 1% (IQR 0–3%) [6]. Subsequent meta-analyses have confirmed transient rates of approximately 25% and permanent rates of 1–5%, depending on the population studied and the definitions applied [7,8,9]. In a large Italian multicenter prospective study of 2631 patients, Puzziello et al. documented transient and permanent hypocalcemia rates of 27.9% and 0.9%, respectively [10], while analysis of the United Kingdom’s national endocrine surgery registry (BAETS) revealed that 23.6% of patients undergoing first-time total thyroidectomy developed biochemical hypocalcemia on postoperative day one (POD1) [11].

Heterogeneity in reported incidence reflects, in part, the absence of a universally accepted definition: studies have variously used 6-month and 12-month thresholds to distinguish transient from permanent hypoPTH [12,13]. The revised 2025 European Society of Endocrinology (ESE) Clinical Practice Guideline defines permanent hypoPTH as persistence beyond 12 months, acknowledging that recovery may still occur thereafter [14,15]. Permanent disease carries a substantial long-term burden—lifelong calcium and active vitamin D supplementation, elevated risks of nephrolithiasis, renal insufficiency and cardiovascular morbidity [16], and significant functional impairment reported by patients [17]—and early stratification between recoverable and chronic forms is therefore central to perioperative management.

Established risk factors include patient-related (female sex, Graves’ disease, autoimmune thyroiditis, and vitamin D deficiency) [6,7,18,19] and surgical determinants (extent of resection, surgeon experience, the number of parathyroid glands preserved in situ, and inadvertent parathyroidectomy) [6,9,20]. The role of prophylactic central neck dissection (CND) is contested: observational data suggest higher hypoPTH rates with bilateral CND [21,22], but the only randomized trial in clinically node-negative papillary thyroid carcinoma (PTC) showed no significant difference in postoperative PTH [23], and a meta-analysis of five RCTs reported a non-significant trend (relative risk [RR] 1.48; 95% confidence interval [CI] 0.73–2.97) [24]. Thyroid gland weight has been proposed as an independent preoperative predictor, with most prior series associating heavier glands with higher risk [19,25], a directionality our data unexpectedly challenge.

The American Thyroid Association (ATA) statement on post-surgical hypoPTH recommends early postoperative PTH-based algorithms for risk stratification, identifying a PTH level below 15 pg/mL as a threshold for increased risk [4]. Studies of POD1 biochemical surveillance have converged on PTH cut-offs in the 10–15 pg/mL range and have shown that combined POD1 calcium and PTH models further improve discriminative ability [26,27,28,29,30,31]; the relative merits of absolute cut-offs versus percent-change criteria are further discussed in detail below (Section 4).

Percent-change metrics—relative decline from preoperative baseline rather than absolute postoperative values—may add predictive information by normalizing for inter-individual variation [32,33,34]. Whether they offer reliable advantages over absolute POD1 thresholds remains incompletely characterized, with few studies comparing the two approaches in a single cohort or examining whether benign and malignant pathology produce different biochemical trajectories.

The present preliminary single-center study has one primary and three secondary, exploratory aims. The primary aim is to evaluate the discriminative performance of POD1 calcium, POD1 PTH, and their combination for the early identification of transient hypoPTH following total thyroidectomy. Because both POD1 calcium and POD1 PTH are constituent variables of the operational definition of transient hypoPTH, this primary aim is by design a confirmatory diagnostic exercise rather than a fully independent predictive one; we therefore complement it with a sensitivity analysis evaluating the same biomarkers against decoupled clinical endpoints (severe biochemical hypocalcemia defined on POD1 calcium alone and permanent hypoPTH). The secondary, exploratory aims are: (i) to identify independent preoperative predictors of transient hypoPTH—with particular attention to thyroid gland weight; (ii) to compare absolute POD1 values against percent-change metrics (ΔCa and ΔPTH); and (iii) to explore the influence of histological malignancy and CND on biochemical decline. The findings reported here reflect the experience of a single academic center during 2023–2025, are intended as preliminary, and are framed to support a planned multicenter prospective extension.

## 2. Materials and Methods

### 2.1. Study Design and Patient Selection

This was a retrospective cohort study conducted at a single academic tertiary referral center. The study included all consecutive patients who underwent total thyroidectomy between 1st of January 2023 and 31st of December 2025. The study protocol was approved by the Institutional Ethics Committee of the Pius Brinzeu Clinical Emergency County Hospital Timisoara (approval no. 189/3 February 2026). Due to the retrospective nature of the study, the requirement for individual informed consent was waived. The study was conducted in accordance with the Declaration of Helsinki and reported following the STROBE (Strengthening the Reporting of Observational Studies in Epidemiology) guidelines.

**Figure 1:**
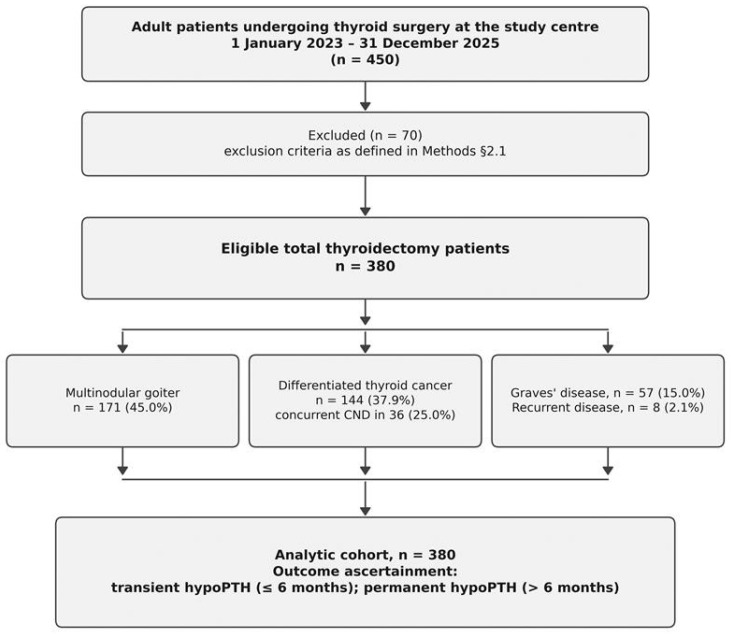
Flow diagram of patient selection. Of 450 adult patients undergoing thyroid surgery at the study center between 1 January 2023 and 31 December 2025, 70 were excluded according to the criteria defined in the Section 2 (Section 2.1), yielding 380 eligible total thyroidectomy patients who formed the analytic cohort. Surgical indications were multinodular goiter (n = 171; 45.0%), differentiated thyroid cancer (n = 144; 37.9%; concurrent CND in 36 of these, 25.0%), Graves’ disease (n = 57; 15.0%) and recurrent disease (n = 8; 2.1%). Outcomes were ascertained as transient hypoPTH (≤6 months postoperatively) and permanent hypoPTH (>6 months postoperatively). CND = central neck dissection; eGFR = estimated glomerular filtration rate; hypoPTH = hypoparathyroidism; POD1 = postoperative day 1.

Eligibility was defined a priori. Inclusion criteria were: (i) age ≥ 18 years; (ii) total thyroidectomy performed at the study center between 1 January 2023 and 31 December 2025; (iii) availability of preoperative and POD1 serum total calcium and intact (i) PTH measurements; and (iv) availability of clinical follow-up to at least six months postoperatively. Exclusion criteria were: (i) thyroid lobectomy or sub-total thyroidectomy as the index operation; (ii) completion thyroidectomy performed for reasons other than thyroid disease recurrence; (iii) pre-existing hypoPTH of any etiology; (iv) chronic kidney disease (eGFR < 60 mL/min/1.73 m^2^); (v) any other documented disorder of calcium metabolism (primary hyperparathyroidism, sarcoidosis, or malabsorptive disease); and (vi) incomplete preoperative or POD1 biochemistry. Concurrent CND, performed only in patients with differentiated thyroid cancer, was not an exclusion. A STROBE-style flow diagram of inclusion and exclusion is provided below, as Figure 1. After application of these criteria, 380 consecutive patients formed the analytic cohort. Surgical indications comprised multinodular goiter (n = 171; 45.0%), differentiated thyroid cancer (n = 144; 37.9%), Graves’ disease (n = 57; 15.0%) and recurrent thyroid disease (n = 8; 2.1%); 36 patients (9.5%), all with differentiated thyroid cancer, underwent concurrent CND.

### 2.2. Perioperative Assessment and Surgical Technique

Preoperative laboratory evaluation included serum calcium (mg/dL) and iPTH (pg/mL) measurements obtained within 24 h prior to surgery. Postoperative serum calcium and iPTH levels were measured on postoperative day 1 (POD1), drawn at approximately 06:00–08:00. Serum calcium was measured using an automated colorimetric assay (reference range: 8.5–10.5 mg/dL), and iPTH was measured by electrochemiluminescence immunoassay (reference range: 15–65 pg/mL).

All procedures were performed under general anesthesia using a standard cervical collar incision (Kocher incision). Total extracapsular thyroidectomy was performed with meticulous identification and preservation of the parathyroid glands and recurrent laryngeal nerves. Intraoperative neuromonitoring (IONM) of the recurrent laryngeal nerve was not used during the study period; the nerve was identified and preserved by visual anatomical dissection in all cases. Parathyroid glands were identified intraoperatively by visual characteristics alone—color (orange–yellow/tan–brown), texture, shape, and anatomical relationship to the recurrent laryngeal nerve and inferior thyroid artery. Near-infrared autofluorescence (NIRAF) and indocyanine green (ICG) angiography were not available, and intraoperative frozen-section pathology was not used to confirm parathyroid tissue. Hemostasis was achieved using the LigaSure™ bipolar vessel-sealing system (Medtronic, Minneapolis, MN, USA), which is the standard energy device employed at our institution for thyroidectomy. Suture ligation and bipolar coagulation were used for vessels and tissues unsuitable for advanced energy-device application. CND (level VI), when indicated, was performed ipsilateral to the primary malignancy. Autotransplantation of inadvertently devascularized parathyroid glands into the sternocleidomastoid muscle was performed when intraoperative devascularization was recognized.

Procedures were performed by a single senior endocrine surgeon (≥5 years post-fellowship specialist experience; n = 347; 91.3%) or by a supervised surgical resident with the senior surgeon scrubbed in for parathyroid identification and inferior-pole dissection (n = 33; 8.7%); the annual institutional volume was approximately 25 thyroidectomies per surgeon. Because case allocation between the two operators was non-random and the denominators are highly imbalanced, senior-vs-resident comparisons are presented descriptively rather than as a causal contrast.

### 2.3. Definitions and Outcome Measures

The primary outcome measures were the incidence of transient and permanent postoperative hypoPTH. Transient hypoPTH was defined as a serum iPTH level below the lower limit of normal (<15 pg/mL) on POD1 and/or symptomatic hypocalcemia (serum calcium <8.0 mg/dL), requiring oral calcium and active vitamin D supplementation, with biochemical and clinical resolution within six months postoperatively. Permanent hypoPTH was defined as the persistence of subnormal PTH levels and/or the need for ongoing calcium and vitamin D supplementation beyond six months after surgery. Patients meeting the criteria for permanent hypoPTH were, by definition, also classified as having had transient hypoPTH. This six-month threshold was applied as it was the prevailing standard at the time of data collection; however, the revised 2025 ESE guideline has since extended the definition of chronic hypoPTH to more than 12 months post-surgery [15], and this discrepancy is addressed in the Discussion. Sensitivity analysis applying a 12-month threshold was not feasible given the available follow-up data, and this represents a limitation.

Standard institutional postoperative follow-up consisted of surgical review at one week and one month, with concurrent referral to the endocrinology service for ongoing management of thyroid hormone replacement and, when relevant, calcium and active vitamin D supplementation. Beyond three to six months, ongoing biochemical surveillance was performed by endocrinology services, frequently at the patient’s referring center rather than our institution; complete prospective biochemical data at 12 months were therefore not systematically available within our surgical electronic record, and a 12-month outcome could not be applied to this cohort. This is acknowledged in the limitations.

Secondary outcome measures included operative time (minutes), length of hospital stay (days), POD1 serum calcium and PTH levels, and the percent change in calcium (ΔCa) and PTH (ΔPTH) from preoperative to POD1 values. The percent change was calculated as [(POD1 value − preoperative value)/preoperative value] × 100 and was examined as an exploratory outcome measure to normalize for inter-individual baseline variation.

### 2.4. Data Collection and Statistical Analysis

Data were retrospectively extracted from electronic medical records and operative reports. The following variables were recorded: patient demographics (age and sex), surgical indication, presence of concomitant Hashimoto thyroiditis, malignancy status, thyroid specimen weight (grams), performance of CND, surgeon seniority (senior surgeon vs. supervised resident), operative time, preoperative and POD1 serum calcium and iPTH levels, and length of hospital stay. No missing data were identified across any variable.

As a retrospective consecutive-patient study, no a priori power calculation was performed. Sample size was assessed post hoc against the conventional events-per-variable (EPV) heuristic [35,36]: with 132 transient hypoPTH events and ten candidate predictors, EPV was 13.2, supporting stable multivariable estimation. Multivariable analysis was not feasible for permanent hypoPTH (n = 11 events, EPV = 1.1), and this outcome is reported descriptively only.

Continuous variables were tested for normality using the Shapiro–Wilk test. As most continuous variables exhibited non-normal distributions, data are expressed as the median with IQR. Categorical variables are presented as frequencies and percentages.

Univariate comparisons between groups were performed using the Mann–Whitney U test for continuous variables and the chi-square test or Fisher’s exact test (when expected cell counts were below 5) for categorical variables. For Table 1 (see Results), in addition to an overall 4 × 2 χ^2^ across surgical indications, per-indication *p*-values were computed comparing each indication versus all other indications combined (Fisher’s exact test); these are reported as exploratory and unadjusted for multiple testing.

Multivariable binary logistic regression was performed to identify independent pre- and intraoperative predictors of transient hypoPTH. All clinically relevant pre- and intraoperative covariates were entered into the model regardless of univariate significance (forced-entry method). In subgroup analyses where quasi-complete separation precluded estimation of specific covariates (e.g., senior surgeon status in the malignant subgroup), the affected variable was excluded from the model. Model calibration was assessed using the Hosmer–Lemeshow goodness-of-fit test, calibration slope, and Brier score. Multicollinearity was evaluated by computing variance inflation factors (VIFs) for all predictors; VIF values below 5 were considered acceptable. Odds ratios (ORs) with 95% CIs were calculated. Due to the low event count for permanent hypoPTH (n = 11), multivariable logistic regression was not feasible for this outcome, and results are reported descriptively.

Multivariable linear regression was used to identify independent predictors of continuous postoperative outcomes, including POD1 serum calcium, POD1 PTH, length of hospital stay, and the exploratory percent-change metrics (ΔCa and ΔPTH). Model performance was assessed using the coefficient of determination (R^2^) and the overall F-test. Unstandardized regression coefficients (β) with 95% CI are reported. Correlations between continuous variables were assessed using Spearman’s rank correlation coefficient (ρ).

The discriminative ability of POD1 calcium, POD1 PTH, ΔCa and ΔPTH was assessed by ROC analysis with Youden-index cut-offs. Because POD1 calcium and POD1 PTH are constituents of the composite outcome definition, a pre-specified sensitivity analysis re-evaluated POD1 PTH and ΔPTH against two PTH-independent outcomes—POD1-calcium-defined severe hypocalcemia (POD1 Ca < 8.0 mg/dL) and permanent hypoPTH; preoperative thyroid weight was evaluated against permanent hypoPTH as an internal control. A combined POD1 calcium + PTH logistic model was compared with each single-biomarker model by bootstrap AUC difference (2000 iterations) and net reclassification improvement (NRI). As pre-specified robustness checks, multilevel linear models with surgeon-level random intercepts and a generalized estimating equation (GEE) model with exchangeable correlation were fitted for continuous and binary outcomes respectively (reported in the Section 3).

Pre-specified subgroup analyses were performed, stratifying the cohort by histological malignancy status (benign vs. malignant) and by performance of CND (CND vs. no CND). Within each subgroup, univariate comparisons and separate multivariable logistic-regression models were fitted to explore potential differences in predictor profiles. Hashimoto thyroiditis was excluded from the malignant subgroup model when no malignant case had coexisting Hashimoto thyroiditis (zero-cell problem).

Statistical analyses were performed using SPSS version 28.0 (IBM Corp., Armonk, NY, USA) for descriptive statistics, univariate comparisons, logistic and linear regression, and ROC analysis. R version 4.3 (R Foundation for Statistical Computing, Vienna, Austria) was used for bootstrap AUC comparisons, NRI calculations, multilevel modeling (lme4 package), and GEE (geepack package). All tests were two-tailed, and a *p*-value of <0.05 was considered statistically significant.

## 3. Results

### 3.1. Patient Characteristics and Baseline Data

The analytic cohort comprised 380 consecutive patients (193 males, 50.8%; median age 53 years, IQR 38–69). Detailed demographic, surgical and biochemical characteristics—including surgical indication, Hashimoto thyroiditis status, histological malignancy, performance of CND, and operator—are presented in Table 1, stratified by transient hypoPTH status. No missing data were identified across any variable.

The median operative time was 52.9 min (IQR 42.4–64.1), and the median thyroid gland weight was 45.0 g (IQR 31.0–58.7). Baseline preoperative calcium was 9.31 mg/dL (IQR 9.11–9.49), and preoperative PTH was 39.8 pg/mL (IQR 32.9–47.6). The complete demographic breakdown stratified by transient hypoPTH status is presented in Table 1. Per-indication univariate analyses (each indication versus all others; Fisher’s exact test) showed no significant association of transient hypoPTH with any specific surgical indication: multinodular goiter (33.3% vs. 36.4%; *p* = 0.665), differentiated thyroid cancer (38.9% vs. 32.2%; *p* = 0.222), Graves’ disease (29.8% vs. 35.6%; *p* = 0.452), or recurrent disease (25.0% vs. 34.9%; *p* = 0.719). The overall 4 × 2 chi-square across indications was non-significant (χ^2^ = 2.19, df = 3, *p* = 0.535). These exploratory comparisons are unadjusted for multiple testing.

### 3.2. Postoperative Biochemical Outcomes

On postoperative day 1 (POD1), the median serum calcium was 8.48 mg/dL (IQR 7.89–8.78), representing a mean decline of 0.95 mg/dL (mean percent change of −10.0%) from preoperative values. The median POD1 PTH was 23.6 pg/mL (IQR 11.6–33.2), corresponding to a mean decline of 17.1 pg/mL (−38.5%). Transient hypoPTH occurred in 132 patients (34.7%), while permanent hypoPTH developed in 11 (2.9%). The median length of hospital stay was 2.3 days (IQR 1.8–3.1).

**Figure 2:**
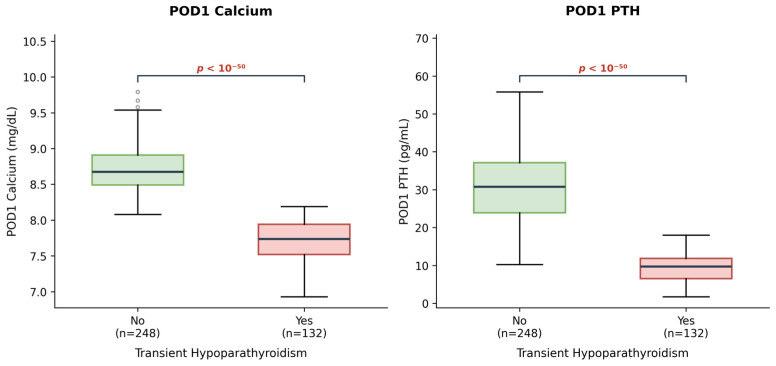
Distribution of postoperative day 1 (POD1) calcium and parathyroid hormone (PTH) values stratified by transient hypoparathyroidism status. Box plots show median (horizontal line), interquartile range (box), and whiskers extending to 1.5 × IQR. Outliers are shown as individual points.

Patients with transient hypoPTH had markedly lower POD1 calcium (median 7.74 vs. 8.68 mg/dL, *p* < 10^−57^) and POD1 PTH (median 9.67 vs. 30.7 pg/mL, *p* < 10^−56^) compared to unaffected patients (Figure 2). Patients who developed transient hypoPTH also had significantly longer hospital stays (median 3.2 vs. 2.1 days, *p* < 10^−24^).

Permanent hypoPTH occurred in 11 patients (2.9%), all of whom were initially classified within the transient hypoPTH group. These patients had markedly lower POD1 calcium (median 7.22 vs. 8.49 mg/dL, *p* < 0.001) and POD1 PTH (median 3.97 vs. 24.1 pg/mL, *p* < 0.001) compared to the remaining cohort. The median age was higher (65 vs. 52 years, *p* = 0.051), though this did not reach significance. All 11 cases were operated by the senior surgeon. The predominant indication was differentiated thyroid cancer (6/11, 54.5%). Hospital stay was longer (median 4.0 vs. 2.3 days). Due to the low event count (n = 11), multivariable logistic regression was not feasible (singular matrix), and results should be interpreted descriptively.

### 3.3. Univariate Predictors of Transient Hypoparathyroidism

Among pre- and intraoperative variables, three factors showed significant univariate associations with transient hypoPTH. Patients who developed transient hypoPTH had a significantly lower thyroid gland weight (median 39.5 vs. 47.5 g, *p* = 0.003) and shorter operative times (median 50.4 vs. 54.0 min, *p* = 0.018). Additionally, a higher proportion of cases performed by a senior surgeon developed transient hypoPTH (96.2% vs. 88.7%, *p* = 0.023), though this association reflects case allocation and the highly imbalanced denominator rather than a causal effect of surgical seniority: more complex resections were preferentially assigned to the senior surgeon, who performed 91.3% of all procedures, and the variable does not survive multivariable adjustment (see Section 3.4). The comparison is presented for transparency only and is not interpreted as evidence regarding surgeon performance.

No significant univariate associations were observed for age (*p* = 0.317), sex (*p* = 0.456), surgical indication (*p* = 0.535), Hashimoto thyroiditis (*p* = 1.000), malignancy (*p* = 0.224), CND (*p* = 1.000), preoperative calcium (*p* = 0.456), or preoperative PTH (*p* = 0.220).

### 3.4. Multivariable Logistic Regression: Transient Hypoparathyroidism

**Figure 3:**
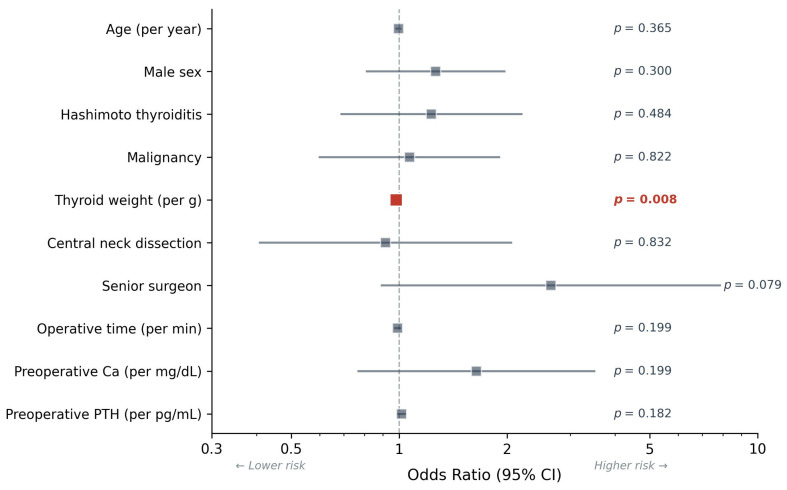
Forest plot of odds ratios from multivariable logistic regression for transient hypoparathyroidism. Red squares indicate statistically significant predictors (*p* < 0.05). The dashed vertical line represents OR = 1 (no effect). Horizontal lines represent 95% confidence intervals.

On multivariable logistic regression adjusting for all pre- and intraoperative covariates, thyroid gland weight was the only independent predictor of transient hypoPTH (Figure 3). Each gram increase in thyroid weight was associated with a 1.8% reduction in the odds of transient hypoPTH (OR 0.982, 95% CI 0.969–0.995, *p* = 0.008). The overall model was statistically significant (likelihood ratio test, *p* = 0.014), with a pseudo-R^2^ (McFadden) of 0.045. The pseudo-R^2^ of 0.045 is low and confirms that preoperative variables, considered together, account for only a small fraction of the variability in early postoperative parathyroid function. Thyroid gland weight reaches statistical significance and is informative as a risk-modulating factor, but it should not be interpreted as a stand-alone screening tool; the clinically relevant decision threshold continues to lie with POD1 biochemical surveillance.

Senior surgeon status showed a trend toward higher odds of transient hypoPTH (OR 2.65, 95% CI 0.895–7.848, *p* = 0.079), but this did not reach statistical significance. No other pre- or intraoperative variable was independently associated with the outcome in the adjusted model. Variance inflation factors for all predictors ranged from 1.02 to 1.68, indicating no problematic multicollinearity.

The preoperative logistic regression model demonstrated adequate calibration (Hosmer–Lemeshow χ^2^ = 7.33, df = 8, *p* = 0.501; calibration slope = 1.00; Brier score = 0.214). Despite good calibration, the low overall discrimination (pseudo-R^2^ = 0.045) confirms that preoperative factors have limited prognostic utility compared to postoperative day 1 biomarkers.

### 3.5. Multivariable Linear Regression: Continuous Outcomes

Multivariable linear regression for POD1 calcium identified two independent predictors (Table 2): thyroid weight was positively associated with POD1 calcium (β = +0.005 per gram, 95% CI 0.002–0.009, *p* = 0.003), and preoperative calcium showed a paradoxical inverse association (β = −0.226 per mg/dL, 95% CI −0.421 to −0.031, *p* = 0.023). The overall model explained 4.8% of the variance in POD1 calcium (adjusted R^2^ = 0.022, F-test, *p* = 0.048).

**Figure 4:**
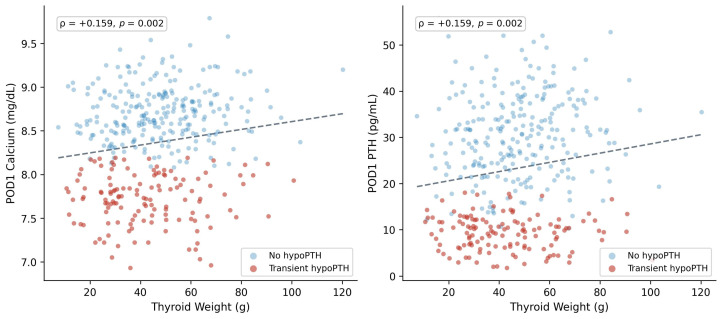
Scatter plots of thyroid gland weight vs. POD1 calcium (**left**) and POD1 PTH (**right**), with points colored by transient hypoparathyroidism status. Dashed line represents the overall linear trend. Spearman correlation coefficients are shown. Note the clustering of transient hypoparathyroidism cases (red) among smaller thyroid glands with lower POD1 biochemical values.

For POD1 PTH (Table 2), only thyroid weight reached significance (β = +0.103 per gram, 95% CI 0.027–0.178, *p* = 0.008), with the model explaining 4.9% of variance (adjusted R^2^ = 0.024, F-test, *p* = 0.041). The multivariable model for length of stay was not statistically significant (R^2^ = 0.025, F-test, *p* = 0.488), though senior surgeon status trended toward longer stays (β = +0.40 days, *p* = 0.051). The relationship between thyroid weight and postoperative biochemical parameters is visualized in Figure 4.

### 3.6. Discriminative Performance of Postoperative Day 1 Biomarkers

**Figure 5:**
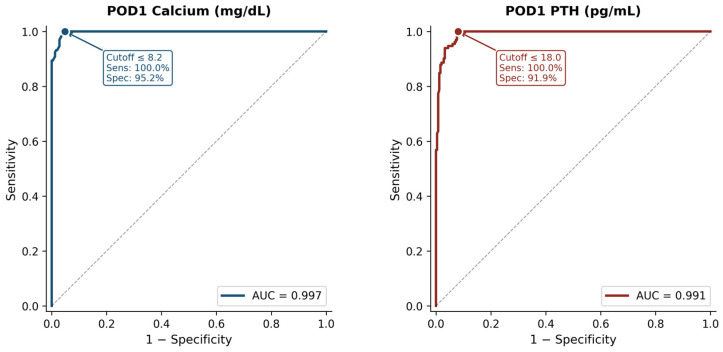
Receiver operating characteristic (ROC) curves for POD1 calcium (**left**) and POD1 PTH (**right**) in predicting transient hypoparathyroidism. Filled circles indicate the Youden-optimal cut-off point. The dashed diagonal line represents the reference line of no discrimination (i.e., the expected performance of a test no better than chance, AUC = 0.50). Both biomarkers demonstrated near-perfect discrimination (AUC > 0.99).

ROC analysis is presented in Figure 5 and Table 3. Because POD1 calcium and POD1 PTH are constituent variables of the composite definition of transient hypoPTH, the discriminative metrics reported in this subsection partly reflect the internal consistency of the outcome definition rather than independent predictive performance; the decoupled-outcome sensitivity analysis in Section Sensitivity Analysis with Decoupled Outcomes provides the more conservative estimate of the true biochemical signal, and the reader is referred there for the interpretive headline. With this caveat foregrounded, for the composite outcome, POD1 calcium yielded an AUC of 0.997 (95% CI 0.994–1.000), with a Youden-optimal cut-off of ≤8.19 mg/dL (sensitivity 100%, specificity 95.2%); at the more conservative cut-off of ≤8.0 mg/dL, sensitivity was 82.6% with 100% specificity. POD1 PTH yielded an AUC of 0.991 (95% CI 0.985–0.998), with a Youden-optimal cut-off of ≤18.02 pg/mL (sensitivity 100%, specificity 91.9%); at the rounded cut-off of ≤15.0 pg/mL, sensitivity was 93.9% and specificity 95.6%.

A combined logistic-regression model incorporating both POD1 calcium and PTH yielded an AUC of 1.000 (sensitivity 100.0%, specificity 98.8%); bootstrap differences in AUC versus the single-biomarker models were small but statistically distinguishable (ΔAUC vs. calcium = +0.002, *p* = 0.010; vs. PTH = +0.009, *p* < 0.001), and NRI over the POD1 calcium ≤ 8.19 mg/dL cut-off was +0.036, driven by correct downward reclassification of 12 non-events. As noted above, the AUC of 1.000 partly reflects the fact that the combined biomarker carries the full information of the outcome definition; the modest but consistent incremental NRI nevertheless suggests genuine complementary information between calcium and PTH, supporting their joint use for early postoperative biochemical surveillance.

Robustness checks for surgeon-level clustering: Multilevel linear models with a random intercept for surgeon, and a GEE model with an exchangeable correlation structure for the binary outcome, were fitted as pre-specified robustness checks; intraclass correlation coefficients were negligible (ICC ≈ 0 for POD1 calcium; 0.035 for POD1 PTH; 0.060 for length of stay), and fixed-effect estimates were essentially identical to the standard regression models in magnitude and significance. The GEE estimates corroborated thyroid gland weight as the principal predictor of transient hypoPTH (OR 0.983, 95% CI 0.978–0.988, *p* < 0.001); incidental associations with operative time, malignancy and preoperative calcium that emerged under GEE most likely reflect improved standard-error efficiency rather than substantively different inference and are not interpreted as primary findings.

#### Sensitivity Analysis with Decoupled Outcomes

Because POD1 calcium and POD1 PTH form part of the composite operational definition of transient hypoPTH, their use as predictors of that same definition introduces partial circularity. To probe whether the central biochemical signal survives this circularity, we re-evaluated the discriminative performance of POD1 PTH and ΔPTH against two outcomes that do not include PTH in their definition: (i) severe biochemical hypocalcemia defined on POD1 calcium alone (POD1 Ca < 8.0 mg/dL; n = 107 events) and (ii) permanent hypoPTH (n = 11 events). The discriminative performance of preoperative thyroid gland weight against permanent hypoPTH was also evaluated as an internal control.

POD1 PTH retained excellent discrimination for severe biochemical hypocalcemia (AUC = 0.943) and for permanent hypoPTH (AUC = 0.976). ΔPTH retained excellent discrimination for severe biochemical hypocalcemia (AUC = 0.933) and for permanent hypoPTH (AUC = 0.956). Thyroid gland weight, by contrast, did not discriminate permanent hypoPTH (AUC = 0.46), consistent with its restricted role as a risk modifier for a transient rather than permanent disease. Together, these analyses indicate that the central finding—strong biochemical discrimination of clinically relevant parathyroid is compromised by POD1 PTH and its relative decline—is robust to the partial circularity of the original composite endpoint, while the headline AUCs of 0.991–1.000 are best interpreted as a confirmation of the composite definition rather than as evidence of a fully independent diagnostic test. Detailed results are provided in Table 4.

### 3.7. Subgroup Analysis

#### 3.7.1. Malignant vs. Benign Disease

**Figure 6:**
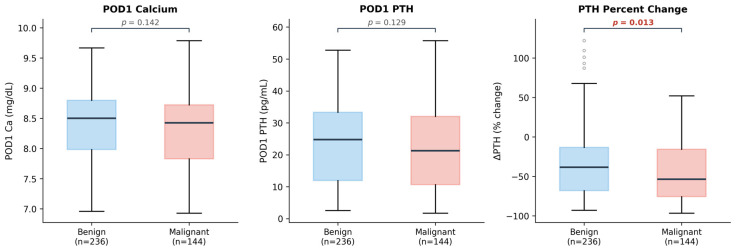
Comparison of POD1 calcium, POD1 PTH, and PTH percent change between benign and malignant cases. The significant difference in PTH percent change (*p* = 0.013) indicates greater relative parathyroid suppression in malignant disease despite comparable absolute POD1 levels.

The cohort was stratified by histological malignancy status (benign: n = 236, malignant: n = 144) to assess whether the relationship between preoperative factors and postoperative outcomes differed by disease type (Table 5, Figure 6). The rate of transient hypoPTH was numerically higher in the malignant subgroup (38.9% vs. 32.2%) but did not reach statistical significance (*p* = 0.224). Permanent hypoPTH rates were similarly comparable (4.2% vs. 2.1%, *p* = 0.346). Notably, malignant cases had significantly smaller thyroid glands (median 35.5 vs. 50.5 g, *p* < 0.001), and all 36 CNDs were in the malignant group.

The only biochemical outcome that differed significantly between groups was the percent change in PTH (ΔPTH): malignant cases showed a greater median decline (−53.7% vs. −38.5%, *p* = 0.013), suggesting more substantial parathyroid functional compromise despite comparable absolute POD1 values. Absolute POD1 calcium and PTH levels, length of stay, and calcium percent change did not differ significantly between groups.

**Figure 7:**
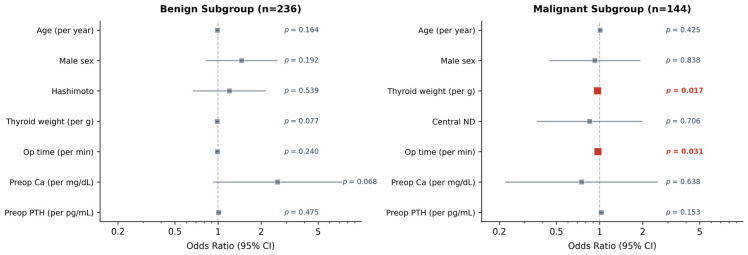
Forest plots of subgroup logistic regression for transient hypoparathyroidism. (**Left**): benign subgroup (no significant predictors). (**Right**): malignant subgroup (thyroid weight and operative time are significant). Red squares and bold labels indicate *p* < 0.05.

Within-subgroup multivariable logistic regression (Figure 7) revealed distinct predictor profiles. In the benign subgroup, no individual predictor reached statistical significance, though thyroid weight (OR 0.986, *p* = 0.077) and preoperative calcium (OR 2.601, *p* = 0.068) showed trends. The overall model was not significant (LR *p* = 0.109). In the malignant subgroup, both thyroid weight (OR 0.967, 95% CI 0.940–0.994, *p* = 0.017) and operative time (OR 0.975, 95% CI 0.953–0.998, *p* = 0.031) were independently significant, with the overall model reaching significance (pseudo-R^2^ = 0.076, LR *p* = 0.042). CND was not an independent predictor (OR 0.851, *p* = 0.706). Hashimoto thyroiditis was excluded from the malignant subgroup model as no malignant case had co-existing Hashimoto thyroiditis.

#### 3.7.2. Central Neck Dissection

**Figure 8:**
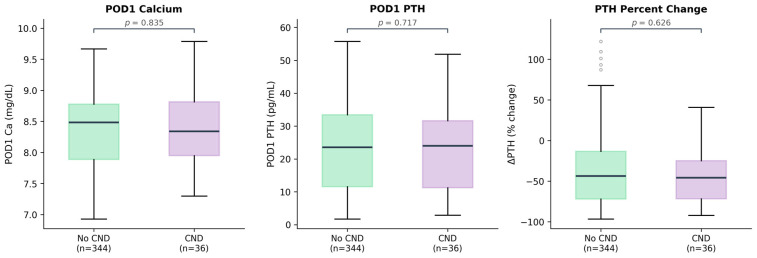
Comparison of POD1 calcium, POD1 PTH, and PTH percent change between patients with and without central neck dissection. No significant differences were observed for any outcome (all *p* > 0.6).

CND was performed in 36 patients (9.5%), all of whom had a malignant disease (Table 6, Figure 8). The CND group had significantly smaller thyroid glands (median 33.4 vs. 45.5 g, *p* = 0.002), consistent with their malignant histology. Despite the additional surgical dissection, CND did not significantly increase the rate of transient hypoPTH (36.1% vs. 34.6%, *p* = 1.000) or any other postoperative outcome. POD1 calcium (*p* = 0.835), POD1 PTH (*p* = 0.717), calcium and PTH percent change (*p* = 0.718 and 0.626, respectively), and length of stay (*p* = 0.653) were all comparable between groups.

No permanent hypoPTH occurred in the CND group (0/36 vs. 11/344 in non-CND, *p* = 0.609), though the sample size precludes meaningful conclusions. Within the CND subgroup (n = 36), operative time showed a borderline trend as a predictor of transient hypoPTH (median 51.0 vs. 56.1 min, *p* = 0.075), while thyroid weight, age, preoperative calcium, and PTH did not differ between affected and unaffected patients (all *p* > 0.35). The limited statistical power of this subgroup (n = 36, with only 13 events) must be acknowledged.

### 3.8. Exploratory Analysis: Biochemical Percent Change (ΔCa and ΔPTH)

**Figure 9:**
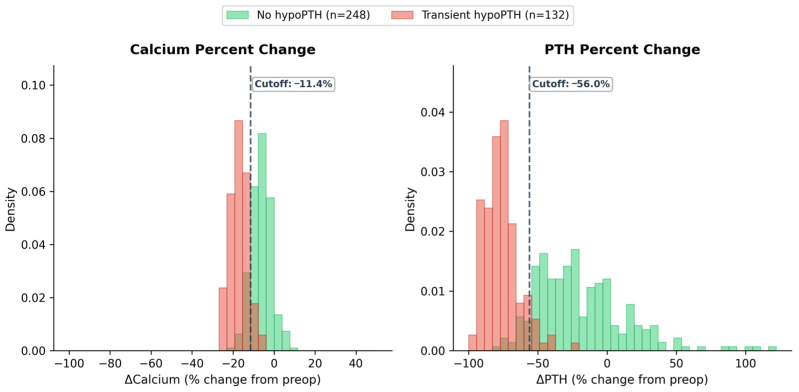
Distribution of percent change in calcium (**left**) and PTH (**right**), stratified by transient hypoparathyroidism status. Dashed lines indicate the Youden-optimal cut-off. Note the clear bimodal separation, particularly for ΔPTH.

The percent change in calcium (ΔCa) and PTH (ΔPTH) from preoperative to POD1 values were examined as exploratory outcome measures that normalize for inter-individual baseline variation. The median ΔCa was −9.6% (IQR −15.0 to −5.1), and the median ΔPTH was −43.8% (IQR −72.1 to −13.7). Both metrics showed very strong separation between patients with and without transient hypoPTH (Figure 9): ΔCa was −17.4% vs. −6.4% (*p* < 10^−48^), and ΔPTH was −77.2% vs. −25.6% (*p* < 10^−53^).

Multivariable linear regression for ΔCa (Table 7) explained substantially more variance than the absolute POD1 Ca model (R^2^ = 0.276 vs. 0.048), primarily driven by the strong inverse effect of preoperative calcium (β = −12.07 per mg/dL, *p* < 0.001)—patients with higher preoperative calcium experienced proportionally greater declines. Thyroid weight remained independently significant (β = +0.057 per gram, *p* = 0.002). The ΔPTH model (Table 7) also showed a substantially improved fit (R^2^ = 0.210 vs. 0.049), with preoperative PTH as the dominant predictor (β = −1.67 per pg/mL, *p* < 0.001) and thyroid weight again contributing independently (β = +0.251 per gram, *p* = 0.020).

As a discriminative biomarker for transient hypoPTH, ΔPTH achieved an AUC of 0.978 (optimal cut-off ≤−56.0%, sensitivity 93.2%, specificity 93.5%), while ΔCa achieved an AUC of 0.956 (optimal cut-off ≤−11.4%, sensitivity 92.4%, specificity 85.5%). Though slightly lower than the absolute POD1 values (AUC 0.991–0.997), the delta metrics remain excellent discriminators and may offer clinical advantages by accounting for individual baseline variation. Among categorical predictors, malignancy was associated with greater ΔPTH decline (−53.7% vs. −38.5%, *p* = 0.013) and senior surgeon status with greater ΔPTH decline (−45.1% vs. −30.1%, *p* = 0.043).

## 4. Discussion

In this single-center cohort of 380 adult patients undergoing total thyroidectomy, transient hypoPTH occurred in 34.7% and permanent hypoPTH in 2.9% of patients. Three findings frame the present discussion. First, smaller thyroid glands—not larger ones—emerged as the only independent preoperative predictor of transient hypoPTH on multivariable analysis. Second, POD1 calcium and PTH, used in combination, identified patients with transient hypoPTH on the day after surgery with very high discriminative accuracy; however, because these biomarkers are themselves constituents of the operational outcome definition, the composite-outcome AUCs partly reflect internal definitional consistency rather than fully independent predictive performance. Third, patients with a histologically malignant disease exhibited a significantly greater relative PTH decline than patients with a benign disease despite comparable absolute POD1 values, a dissociation that has direct implications for the choice of biochemical surveillance metric in cancer surgery. Each of these observations is discussed against the existing literature below, together with the methodological caveats that limit their generalizability.

The transient hypoPTH rate of 34.7% in this cohort lies within the broadly reported 20–40% range [6,7,8] and is consistent with an academic tertiary setting using a biochemical (rather than symptom-based) definition. High-volume specialist centers have reported substantially lower transient rates (~17%) with comparably low permanent rates [37], underscoring the influence of surgeon expertise.

Our 2.9% permanent hypoPTH rate sits between the figures reported by academic-center meta-analyses (~1–2% [6,7]) and population-based registries (6–12% in the Swedish registry [18,38] and ~7% in the BAETS registry [11]); the gap reflects well-documented selection and reporting biases [12]. Two definitional refinements bear on these numbers. First, our 6-month threshold predates the revised 2025 ESE guideline, which defines chronic hypoPTH at >12 months on the evidence that ~7.5% of patients recover between months 6 and 12 [15]; the 2.9% figure therefore modestly overestimates the truly chronic burden. Second, the 2025 joint consensus statement of the European Society of Endocrine Surgeons, American Association of Endocrine Surgeons, and International Association of Endocrine Surgeons (ESES/AAES/IAES) proposes a minimum reporting dataset for postoperative hypoPTH [39], and our planned multicenter extension has been framed to align with it.

The 37.9% malignancy rate in this cohort, the 9.5% concurrent CND rate, and the 34.7% transient hypoPTH rate are best read together as the case-mix signature of a tertiary referral center. The 2023 third edition of the Bethesda System for Reporting Thyroid Cytopathology (TBSRTC) [40] predicts a 2–7% (average ~4%) malignancy risk for Bethesda II (benign) and 13–30% for Bethesda III (atypia of undetermined significance) nodules; referral-center series have reported real-world malignancy rates of 25–34% in resected Bethesda III and substantially higher rates in Bethesda IV [41,42], bracketing the proportion observed here. The 2015 ATA guideline restricts total thyroidectomy to higher-risk differentiated thyroid carcinoma (DTC), multifocal disease, contralateral nodules, or planned radioactive iodine (RAI) therapy, with lobectomy acceptable for low-risk unifocal cancers ≤ 4 cm [3]; meta-analytic data consistently show higher rates of transient hypocalcemia, recurrent laryngeal nerve palsy and hypoPTH after total than after less-than-total operations [8,43], and incidental parathyroidectomy is more frequent with total resection [43].

This is a trade-off, not an argument against total thyroidectomy where it remains oncologically indicated: the appropriate response is meticulous parathyroid preservation and, where available, adjunctive identification technologies. The proportions reported here reflect exactly this selection pressure—patients submitted to total thyroidectomy at our center in 2023–2025 are predominantly those for whom less-than-total surgery is no longer adequate, and any biochemical risk-stratification protocol must therefore be valid in mixed cohorts rather than only in those operated on a confirmed cancer diagnosis.

Two methodological factors not modelled in this study deserve brief mention. All procedures used the LigaSure bipolar vessel-sealing system: pooled meta-analyses comparing LigaSure and the harmonic scalpel in open thyroidectomy show no significant difference in blood loss, postoperative bleeding, length of stay or postoperative calcium [44,45], with comparable rates of severe bleeding requiring reoperation. A possible harmonic-scalpel advantage in hemostasis during cancer dissection has been reported in single-center series but is not reproduced in pooled randomized data [44]. Because the energy device was not varied across our cohort, no comparative inference is possible.

Retrosternal goiter is encountered in 5–15% of thyroidectomy populations and is uncommonly co-incident with overt thyroid cancer [46]. Radiological characterization of retrosternal extension was not uniformly recorded in our electronic record; we therefore cannot report the precise number of cases with concomitant retrosternal extension and differentiated thyroid carcinoma, nor model retrosternal extension as a predictor. Indications for surgery, predictors of the need for sternotomy (sub-aortic, posterior-mediastinal or sub-carinal extension; a constricting “conical” thoracic-inlet morphology), and the >90% transcervical-delivery rate achievable in modern series are addressed in detail elsewhere [46,47]. Retrosternal extension is a planned variable for the multicenter extension.

The most striking finding of this study is the identification of thyroid gland weight as the only independent preoperative predictor of transient hypoPTH, with an inverse relationship: each gram increase in gland weight reduced the odds of transient hypoPTH by 1.8% (OR 0.982, *p* = 0.008). Smaller glands conferring higher risk is counterintuitive and warrants careful contextualization.

The literature on thyroid weight as a predictor is sparse and directionally inconsistent. A large German multicenter study of 7911 patients explicitly noted how little data exist on specimen weight as a morbidity determinant and found that a thyroid weight > 100 g independently predicted vocal-cord dysfunction and surgical-site infection but could not analyze the relationship with hypoPTH owing to incomplete follow-up [48]. The Sitges-Serra and Karamanakos groups have, by contrast, consistently associated heavier specimens—particularly in Graves’ disease—with protracted and permanent hypoPTH, attributing this to more extensive dissection, longer operative times and more aggressive vascular ligation [19,20,49]. Our data point in the opposite direction, which must be explained on different grounds.

Higher risk with smaller glands likely reflects a mechanism unrelated to surgical complexity. We propose two complementary explanations. First, in smaller glands the parathyroids may lie in closer proximity to the thyroid capsule, narrowing the surgical plane between thyroid and parathyroid tissue and increasing the risk of inadvertent devascularization or mechanical injury during dissection. Second, smaller glands provide fewer anatomical landmarks and less tissue bulk to facilitate visual distinction of parathyroid tissue from surrounding fat, lymph nodes and thyroid parenchyma—and parathyroid identification rates are themselves a critical determinant of outcome. Lorente-Poch et al. demonstrated a strong dose–response relationship using the Parathyroid Glands Remaining In Situ (PGRIS) score: permanent hypoPTH occurred in 16% of patients with PGRIS 1–2, 6.5% with PGRIS 3, and 2.6% with PGRIS 4 (*p* < 0.001) [20]. Any factor that impairs identification—including small gland size—would logically reduce PGRIS and elevate risk.

The pediatric literature is consistent with a context-dependent relationship: Nordenström et al. found no association between specimen weight and hypoPTH in 274 children, with operative time being the sole predictor [50]. The conflicting directionality across adult and pediatric series suggests the relationship may be U-shaped, with both extremes of gland size conferring elevated risk through different mechanisms—large glands via surgical complexity and vascular sacrifice, and small glands via impaired parathyroid identification.

The headline composite-outcome AUCs reported here (0.997 for POD1 calcium, 0.991 for POD1 PTH, and 1.000 combined) are inflated by the structural overlap between predictors and outcome and are not directly comparable with external series. The appropriate benchmark is the decoupled-outcome analysis: against PTH-independent endpoints, POD1 PTH yielded AUCs of 0.943 (severe POD1-calcium-defined hypocalcemia) and 0.976 (permanent hypoPTH), with ΔPTH performing similarly (0.933 and 0.956). These values sit in the upper range of the contemporary literature: the Nagel meta-analysis (188 studies) reported pooled AUCs of 0.94–0.97 for postoperative PTH within 24 h [29]; Noordzij et al. reported 0.97 for 6 h PTH and 0.94 for 1–2 h PTH [51]; Inversini et al. 0.931 for 6 h iPTH [52]; and Kolahdouzan et al. 0.843–0.878 for early iPTH metrics [53]. Published AUCs for POD1 calcium alone are typically lower (from 0.64 [53] to 0.9 for ionized calcium [54]), and combined PTH-plus-calcium models have previously reached 100% sensitivity and specificity in smaller cohorts [55,56].

Read together, these data support the interpretation that POD1 calcium and PTH carry genuinely complementary information about early parathyroid compromise—PTH reflecting glandular injury, and calcium reflecting downstream metabolic consequence with temporal lag [57]—but the composite-outcome AUCs we report should be read as evidence of internal consistency of the outcome definition, not as evidence of an independent diagnostic test. The decoupled-outcome AUCs are the conservative figures suitable for inter-study comparison; the composite figures are not.

Reported POD1 PTH cut-offs converge on 10–15 pg/mL [4,26,27,28,31]—a range consistent with the Youden-optimal 18.0 pg/mL threshold and the more clinically conservative 15.0 pg/mL threshold (sensitivity 93.9%, specificity 95.6%) observed here. Combined calcium-plus-PTH thresholds have repeatedly identified patients safely discharged without supplementation [30,31].

That percent-change metrics (ΔCa and ΔPTH) explained substantially more variance in linear regression (R^2^ 0.21–0.28 vs. 0.05) but yielded slightly lower AUCs for binary classification than absolute POD1 values is not paradoxical. Percent-change metrics, by normalizing to individual baselines, capture a continuous gradient of parathyroid functional compromise—hence the superior R^2^. For the binary task of distinguishing hypo- from euparathyroid states, absolute POD1 values align more directly with the clinically relevant decision threshold, without the variance introduced by heterogeneous baselines.

The broader literature mirrors this duality. Barczyński et al. favored absolute iPTH < 10 pg/mL at 4 h [34]; Lecerf et al. and Van Kinschot et al. favored proportional decline at 4–6 h [58,59]; the Sitges-Serra group reported that an iPTH decline ≥ 62.5% optimally predicted transient hypocalcemia, with declines > 93.7% identifying high risk of permanent disease [60]; and the Nagel meta-analysis endorsed both, recommending absolute thresholds < 10–15 pg/mL alongside relative reductions of ~70% [29]. Absolute metrics offer practical advantages—no preoperative measurement required, simpler workflow, less susceptibility to assay variability—while percent-change metrics may be particularly informative when preoperative PTH is elevated (vitamin D deficiency and secondary hyperparathyroidism), where absolute postoperative values can appear deceptively reassuring. The 2025 ESES/AAES/IAES consensus statement formalizes this complementarity by recommending that both absolute thresholds and relative changes be considered in postoperative risk stratification [37].

The absence of a statistically significant difference in transient hypoPTH with vs. without CND (36.1% vs. 34.6%, *p* = 1.000) aligns with randomized trial evidence that challenges the observational consensus. Observational studies have consistently reported elevated risk with CND: Giordano et al. documented transient hypoPTH rates of 27.7%/36.1%/51.9% for total thyroidectomy alone, ipsilateral CND, and bilateral CND respectively (*p* = 0.014) [21]; Calò et al. confirmed a similar gradient in a multicenter Italian study [22]; and the Zhao meta-analysis of 22 observational studies (6930 patients) reported significantly elevated temporary (OR 2.28) and permanent (OR 1.84) hypoPTH [61].

Randomized controlled trials show a different picture. The Sippel randomized controlled trial (RCT) of 60 clinically node-negative PTC patients found no significant difference in postoperative PTH < 10 pg/mL between prophylactic unilateral CND and thyroidectomy alone (33.3% vs. 24.1%, *p* = 0.57) [23]; the Alsubaie meta-analysis of five RCTs (795 patients) reported a non-significant trend (RR 1.48, 95% CI 0.73–2.97) [24]; and a re-analysis by Sanabria et al. identified a small but significant 3% absolute increase in permanent hypoPTH (risk difference 3%, 95% CI 0–6%) [62].

The discrepancy likely reflects selection bias—observational cohorts disproportionately include bilateral CND in patients with more aggressive disease, larger tumors and extrathyroidal extension—while RCTs have typically studied only ipsilateral prophylactic dissection. Our null finding should be interpreted cautiously given the limited CND subgroup size but is consistent with the emerging RCT-based consensus that prophylactic CND in experienced hands does not dramatically increase hypoPTH risk.

Malignant cases exhibited a significantly greater ΔPTH decline than benign cases (−53.7% vs. −38.5%, *p* = 0.013) despite comparable absolute POD1 PTH values—a novel observation with direct clinical implications. The dissociation suggests that patients with thyroid malignancy experience a greater functional parathyroid insult from surgery even when their postoperative biochemical snapshot looks similar to that of benign cases.

A mechanistic framework is available in the existing literature. Malignancy significantly increases the risk of inadvertent parathyroidectomy (RR 1.60, 95% CI 1.27–2.02) [43]: cancer surgery demands more meticulous dissection of the tracheoesophageal groove, wider margins, and more extensive manipulation of the inferior thyroid artery branches that supply the parathyroid glands. Capsule invasion and extrathyroidal extension are independent predictors of parathyroid compromise [63], and Barrios et al. identified both prophylactic CND (OR 2.68) and therapeutic CND (OR 4.44) as independent predictors of inadvertent parathyroidectomy [63]; concurrent lymph node dissection contributes independently [53].

The comparable absolute POD1 values across malignant and benign diseases, despite divergent percent-change trajectories, may reflect a higher baseline PTH in the malignant group—possibly driven by vitamin D deficiency, which is more prevalent in this population—or compensatory hyperfunction of surviving parathyroid tissue early postoperatively. Either way, this finding exposes a limitation of relying solely on absolute POD1 values for risk stratification in cancer surgery: patients with malignancy may harbor greater parathyroid injury than their absolute postoperative numbers suggest, potentially manifesting as delayed hypocalcemia or slower functional recovery. Percent-change metrics may therefore be particularly valuable in cancer patients, capturing the magnitude of functional decline rather than the static postoperative state.

The paradoxical inverse association between preoperative calcium and POD1 calcium in linear regression is most parsimoniously explained by regression to the mean (RTM), a well-characterized statistical phenomenon in which extreme baseline values tend toward the population mean on subsequent measurement, irrespective of intervention [64,65]. The effect is amplified when serial measurements are imperfectly correlated—precisely the situation when surgery introduces substantial new variance. Higher preoperative calcium values therefore appear to be associated with greater absolute postoperative decline by statistical mechanism alone, and recent meta-analytic identification of preoperative calcium as a transient-hypoPTH risk factor [8] should be interpreted with this caveat in mind; analysis of covariance (ANCOVA)-based change-score analyses are appropriate where baseline-adjusted inference is required [66].

Several established predictors could not be assessed in this retrospective cohort. The PGRIS score—calculated as 4 minus the sum of autotransplanted and inadvertently resected glands—has demonstrated a clear dose–response relationship with parathyroid failure [20,67] and may be the single most important surgical variable; it was not systematically recorded here. Intraoperative parathyroid identification technologies were unavailable: NIRAF reduced postoperative hypocalcemia from 21.7% to 9.1% in the PARAFLUO randomized controlled trial [68] and yielded a pooled odds ratio of 0.56 for transient hypocalcemia in meta-analysis [69], while ICG angiography has demonstrated superior parathyroid identification rates and reduced transient hypocalcemia compared with naked-eye assessment [70]. Future studies incorporating autofluorescence may directly test whether small thyroid glands impair parathyroid identification—the proposed mechanism underlying our weight–risk association.

Preoperative 25(OH) vitamin D was not measured. Meta-analyses confirm that vitamin D deficiency increases hypoPTH risk, with the strongest association at severe deficiency (<15 ng/mL: OR 3.22) [71,72], although the evidence is not entirely consistent—Martín-Román et al. paradoxically reported lower transient hypoPTH in vitamin D-deficient patients, hypothesizing adaptive parathyroid “preconditioning” [73]—and RCTs of preoperative supplementation have yielded mixed results. The 2018 ATA statement nonetheless recommends optimizing preoperative vitamin D levels as a preventive strategy [4].

This study has several important limitations. First, the retrospective single-center design, modest cohort size and academic tertiary case-mix introduce inherent selection and information biases and limit external generalizability; the findings should be regarded as a single-institution preliminary description of a planned multicenter prospective cohort, and case allocation between the senior surgeon and the supervised resident is reported descriptively rather than causally. Second, IONM was not used, and NIRAF, ICG angiography and intraoperative frozen-section pathology were unavailable; parathyroid identification relied on visual assessment alone, and granular data on cases complicated by Hashimoto thyroiditis (where inflammatory perithyroidal lymphadenopathy may obscure identification) were not systematically recorded. Third, preoperative serum 25(OH) vitamin D was not systematically measured and could not be entered into the multivariable model despite robust meta-analytic evidence of its prognostic relevance [71,72]. Fourth, the low event count for permanent hypoPTH (n = 11) precluded multivariable analysis for this most clinically consequential outcome.

Fifth, the 6-month definition of permanent hypoPTH does not align with the revised 2025 ESE recommendation of a 12-month threshold [39]; a 12-month sensitivity analysis was not feasible because complete biochemical follow-up at 12 months was not systematically available within our surgical electronic record, as most patients were managed by referring endocrinology services beyond the early postoperative period. Sixth, the absence of intraoperative PTH measurement and of systematic PGRIS documentation prevents adjustment for what may be the single most important surgical determinant of parathyroid outcome [20,67]. Seventh—and most importantly—POD1 calcium and POD1 PTH are constituent variables of the operational definition of transient hypoPTH; the composite-outcome AUCs (0.99–1.00) therefore reflect internal consistency rather than independent diagnostic performance. The decoupled-outcome sensitivity analysis (Section Sensitivity Analysis with Decoupled Outcomes, Table 4) provides the conservative AUC estimates (0.93–0.98) that survive this circularity, but external prospective validation against PTH-independent endpoints is required before the biomarker combination can be promoted as an independent predictive test.

The absence of intraoperative PTH measurements limits comparison with the extensive literature on immediate postoperative PTH kinetics and prevents assessment of whether real-time PTH monitoring could have improved intraoperative decision-making regarding parathyroid autotransplantation. The lack of systematic PGRIS documentation represents a significant gap, as the number of parathyroid glands preserved in situ has been repeatedly demonstrated to be the most powerful predictor of parathyroid outcomes [49,67]. Additionally, the absence of parathyroid autofluorescence or ICG angiography data precludes evaluation of whether these emerging technologies might mitigate the risks associated with smaller thyroid glands. Finally, as a single academic center study, the generalizability of findings to community practice settings—where the surgeon volume is typically lower and complication rates substantially higher—requires caution [39].

## 5. Conclusions

In this single-center preliminary cohort of 380 adult total-thyroidectomy patients, thyroid gland weight was the sole independent preoperative predictor of transient hypoPTH, with smaller—not larger—glands conferring higher risk. Combined POD1 calcium and PTH provided strong biochemical confirmation of transient hypoPTH on the day after surgery, but because both biomarkers form part of the outcome definition, the composite-outcome AUCs are best read as evidence of internal consistency rather than of independent predictive performance. The decoupled-outcome AUCs (0.93–0.98 for POD1 PTH and ΔPTH against PTH-independent endpoints) provide the more conservative benchmark and support a genuine clinical signal. A clinically relevant secondary observation—patients with a malignant disease showed a significantly greater relative PTH decline than those with a benign disease despite comparable absolute postoperative values—supports the routine inclusion of percent-change metrics in postoperative biochemical surveillance, particularly in cancer surgery. These conclusions reflect a single academic center operating without IONM, NIRAF, ICG angiography, intraoperative frozen-section pathology or systematic preoperative 25(OH) vitamin D measurement and require multicenter prospective validation—incorporating PGRIS documentation, autofluorescence-guided surgery, intraoperative PTH, preoperative 25(OH) vitamin D and the 12-month ESE definition of chronic hypoPTH—before they can inform routine practice elsewhere.

## Figures and Tables

**Table 1:** Patient demographics and clinical characteristics stratified by transient hypoparathyroidism (with per-indication *p*-values).

Variable	Total (n = 380)	No hypoPTH (n = 248)	Transient hypoPTH (n = 132)	*p*-Value
Age, years, median [IQR]	53 [38–69]	55.5 [39.8–70.0]	50.5 [36.8–69.0]	0.317
Male sex, n (%)	193 (50.8)	122 (49.2)	71 (53.8)	0.456
Indication, n (%)				0.535 (overall χ^2^)
Multinodular goiter	171 (45.0)	114 (46.0)	57 (43.2)	0.665
Differentiated thyroid cancer	144 (37.9)	88 (35.5)	56 (42.4)	0.222
Graves disease	57 (15.0)	40 (16.1)	17 (12.9)	0.452
Recurrent disease	8 (2.1)	6 (2.4)	2 (1.5)	0.719
Hashimoto thyroiditis, n (%)	90 (23.7)	59 (23.8)	31 (23.5)	1.000
Malignancy, n (%)	144 (37.9)	88 (35.5)	56 (42.4)	0.224
Central neck dissection, n (%)	36 (9.5)	23 (9.3)	13 (9.8)	1.000
Senior surgeon, n (%)	347 (91.3)	220 (88.7)	127 (96.2)	**0.023**
Thyroid weight, g, median [IQR]	45.0 [31.0–58.7]	47.5 [34.2–59.8]	39.5 [28.2–54.7]	**0.003**
Operative time, min, median [IQR]	52.9 [42.4–64.1]	54.0 [43.2–65.0]	50.4 [41.0–59.8]	**0.018**
Preop Ca, mg/dL, median [IQR]	9.31 [9.11–9.49]	9.29 [9.09–9.48]	9.32 [9.12–9.53]	0.456
Preop PTH, pg/mL, median [IQR]	39.8 [32.9–47.6]	39.0 [32.7–47.1]	41.6 [34.2–48.1]	0.220
POD1 Ca, mg/dL, median [IQR]	8.48 [7.89–8.78]	8.68 [8.49–8.91]	7.74 [7.52–7.94]	**<0.001**
POD1 PTH, pg/mL, median [IQR]	23.6 [11.6–33.2]	30.7 [23.9–37.1]	9.67 [6.49–11.8]	**<0.001**
Length of stay, days, median [IQR]	2.3 [1.8–3.1]	2.1 [1.6–2.6]	3.2 [2.5–3.8]	**<0.001**
Permanent hypoPTH, n (%)	11 (2.9)	0 (0.0)	11 (8.3)	**<0.001**

Values are medians [IQR] or n (%). *p*-values: Mann–Whitney U test for continuous variables; Bold values indicate *p* < 0.05; chi-square or Fisher’s exact test for categorical variables. For surgical indication, an overall 4 × 2 χ^2^ was performed; per-indication *p*-values (each indication vs. all others; Fisher’s exact) are reported as exploratory and unadjusted for multiple testing. Rows with bold *p*-values indicate statistical significance (*p* < 0.05). Abbreviations: hypoPTH = hypoparathyroidism; Ca = calcium; PTH = parathyroid hormone; POD1 = postoperative day 1; IQR = interquartile range; n (%) = number (percentage); g = grams; min = minutes.

**Table 2:** Multivariable linear regression—postoperative day 1: calcium vs. parathyroid hormone.

	POD1 Calcium (R^2^ = 0.048, *p* = 0.048)			POD1 PTH (R^2^ = 0.049, *p* = 0.041)		
Variable	β	95% CI	*p*-Value	β	95% CI	*p*-Value
Age (per year)	+0.001	−0.002 to +0.004	0.435	+0.038	−0.029 to +0.105	0.268
Male sex	−0.037	−0.151 to +0.079	0.533	−1.347	−3.915 to +1.221	0.303
Hashimoto thyroiditis	−0.095	−0.245 to +0.054	0.211	+0.029	−3.315 to +3.373	0.987
Malignancy	−0.044	−0.196 to +0.107	0.566	−0.237	−3.622 to +3.148	0.891
Thyroid weight (per g)	**+0.005**	**+0.002 to +0.009**	0.003 **	**+0.103**	**+0.027 to +0.178**	0.008 **
Central neck dissection	+0.056	−0.159 to +0.271	0.608	+0.536	−4.261 to +5.333	0.826
Senior surgeon	−0.099	−0.334 to +0.136	0.407	−4.311	−9.556 to +0.934	0.107
Operative time (per min)	+0.000	−0.004 to +0.004	0.901	+0.050	−0.041 to +0.140	0.280
Preoperative Ca (per mg/dL)	**−0.226**	**−0.421 to −0.031**	0.023 *	−2.215	−6.569 to +2.138	0.318
Preoperative PTH (per pg/mL)	−0.002	−0.008 to +0.004	0.462	−0.037	−0.168 to +0.095	0.586

β = unstandardized coefficient. Bold values indicate *p* < 0.05 (* = *p* < 0.05, ** = *p* < 0.01). Abbreviations: Ca = calcium; CI = confidence interval; PTH = parathyroid hormone; POD1 = postoperative day 1; g = grams; min = minutes.

**Table 3:** Receiver operating characteristic (ROC) analysis: POD1 biomarker performance for predicting transient hypoparathyroidism.

Biomarker	AUC	Cut-off	Sensitivity	Specificity
POD1 Calcium	0.997	≤8.19 mg/dL *	100.0%	95.2%
		≤8.0 mg/dL	82.6%	100.0%
		≤7.5 mg/dL	24.2%	100.0%
POD1 PTH	0.991	≤18.0 pg/mL *	100.0%	91.9%
		≤15.0 pg/mL	93.9%	95.6%
		≤10.0 pg/mL	54.5%	100.0%

* Youden-optimal cut-off point (sensitivity + specificity − 1). Abbreviations: AUC = area under the curve; PTH = parathyroid hormone; POD1 = postoperative day 1.

**Table 4:** Sensitivity analysis: discriminative performance against decoupled outcomes.

Predictor	Outcome	Events/n	AUC
POD1 PTH	Composite transient hypoPTH (original)	132/380	0.991
POD1 PTH	Severe biochemical hypocalcemia (POD1 Ca < 8.0 mg/dL)	107/380	**0.943**
POD1 PTH	Permanent hypoPTH	11/380	**0.976**
ΔPTH (% change)	Composite transient hypoPTH (original)	132/380	0.978
ΔPTH (% change)	Severe biochemical hypocalcemia (POD1 Ca < 8.0 mg/dL)	107/380	**0.933**
ΔPTH (% change)	Permanent hypoPTH	11/380	**0.956**
Thyroid gland weight (preoperative)	Permanent hypoPTH (internal control)	11/380	0.46

Bold AUC values denote outcomes that are mathematically and clinically independent of the predictor under evaluation. The decoupled-outcome AUCs (0.93–0.98 for POD1 PTH and ΔPTH) confirm that the central biochemical signal is genuine and not an artefact of definitional overlap. Thyroid gland weight does not discriminate permanent hypoparathyroidism, consistent with its role as a risk modifier for a transient but not permanent disease. Abbreviations: hypoPTH = hypoparathyroidism; Ca = calcium; PTH = parathyroid hormone; POD1 = postoperative day 1.

**Table 5:** Postoperative outcomes stratified by histological malignancy status (benign vs. malignant).

Variable	Benign (n = 236)	Malignant (n = 144)	*p*-Value
Transient hypoPTH, n (%)	76 (32.2)	56 (38.9)	0.224
Permanent hypoPTH, n (%)	5 (2.1)	6 (4.2)	0.346
POD1 Ca, mg/dL	8.50 [7.98–8.80]	8.43 [7.83–8.72]	0.142
POD1 PTH, pg/mL	24.8 [12.0–33.3]	21.3 [10.7–32.0]	0.129
ΔCa, % change	−8.5 [−14.7 to −4.7]	−10.8 [−15.5 to −5.7]	0.131
ΔPTH, % change	−38.5 [−68.1 to −13.4]	−53.7 [−75.5 to −15.7]	0.013 *
Length of stay, days	2.3 [1.8–3.0]	2.5 [1.8–3.1]	0.665
Central neck dissection, n (%)	0 (0.0)	36 (25.0)	<0.001 *
Thyroid weight, g	50.5 [37.4–65.0]	35.5 [27.1–45.9]	<0.001 *
Operative time, min	52.8 [43.2–64.2]	53.2 [41.0–63.7]	0.993

Rows with bold *p*-values indicate statistical significance (* = *p* < 0.05). Values are median [IQR] or n (%). *p*-values: Mann–Whitney U for continuous, chi-square/Fisher for categorical. ΔCa and ΔPTH represent percentage (%) change from preoperative values. Abbreviations: hypoPTH = hypoparathyroidism; Ca = calcium; PTH = parathyroid hormone; POD1 = postoperative day 1; IQR = interquartile range; n (%) = number (percentage); g = grams; min = minutes.

**Table 6:** Postoperative outcomes stratified by central neck dissection.

Variable	No CND (n = 344)	CND (n = 36)	*p*-Value
Transient hypoPTH, n (%)	119 (34.6)	13 (36.1)	1.000
Permanent hypoPTH, n (%)	11 (3.2)	0 (0.0)	0.609
POD1 Ca, mg/dL	8.48 [7.89–8.78]	8.34 [7.95–8.82]	0.835
POD1 PTH, pg/mL	23.5 [11.6–33.4]	24.0 [11.3–31.7]	0.717
ΔCa, % change	−9.4 [−15.0 to −5.1]	−11.1 [−15.6 to −5.1]	0.718
ΔPTH, % change	−43.6 [−72.1 to −13.4]	−45.7 [−71.8 to −24.9]	0.626
Length of stay, days	2.3 [1.8–3.1]	2.3 [1.8–2.9]	0.653
Malignancy, n (%)	108 (31.4)	36 (100.0)	<0.001 *
Thyroid weight, g	45.5 [32.5–59.8]	33.4 [24.5–46.0]	0.002 *
Operative time, min	52.6 [42.8–63.7]	55.6 [40.8–65.1]	0.868

Rows with bold *p*-values indicate statistical significance (* = *p* < 0.05). Values are median [IQR] or n (%). All CND cases had malignant histology. No significant differences in any postoperative outcome. ΔCa and ΔPTH represent percentage (%) change from preoperative values. Abbreviations: hypoPTH = hypoparathyroidism; Ca = calcium; PTH = parathyroid hormone; POD1 = postoperative day 1; IQR = interquartile range; n (%) = number (percentage); g = grams; min = minutes.

**Table 7:** Multivariable linear regression: ΔCalcium vs. ΔPTH.

	ΔCalcium—R^2^ = 0.276, *p* < 0.001			ΔPTH—R^2^ = 0.210, *p* < 0.001		
Variable	β	95% CI	*p*-Value	β	95% CI	*p*-Value
Age (per year)	+0.013	−0.019 to +0.046	0.419	+0.106	−0.081 to +0.294	0.265
Male sex	−0.423	−1.660 to +0.814	0.502	−4.741	−11.918 to +2.435	0.195
Hashimoto thyroiditis	−1.025	−2.636 to +0.585	0.211	+1.481	−7.863 to +10.826	0.755
Malignancy	−0.479	−2.109 to +1.152	0.564	+0.218	−9.242 to +9.677	0.964
Thyroid weight (per g)	**+0.057**	**+0.021 to +0.093**	0.002 **	**+0.251**	**+0.040 to +0.462**	0.020 *
Central neck dissection	+0.649	−1.662 to +2.959	0.581	−1.545	−14.950 to +11.861	0.821
Senior surgeon	−1.037	−3.564 to +1.490	0.420	−9.264	−23.923 to +5.394	0.215
Operative time (per min)	+0.005	−0.038 to +0.049	0.810	+0.140	−0.112 to +0.392	0.275
Preoperative Ca (per mg/dL)	**−12.066**	**−14.163 to −9.969**	<0.001 ***	−5.742	−17.910 to +6.425	0.354
Preoperative PTH (per pg/mL)	−0.023	−0.086 to +0.040	0.475	**−1.673**	**−2.041 to −1.305**	<0.001 ***

β = unstandardized coefficient. Bold values indicate statistical significance (* = *p* < 0.05; ** = *p* < 0.01; *** = *p* < 0.001). Abbreviations: Ca = calcium; CI = confidence interval; PTH = parathyroid hormone; g = grams; min = minutes. ΔCa and ΔPTH represent percentage (%) change from preoperative values.

## Data Availability

Data available on request.

## Abbreviations

The following abbreviations are used in this manuscript:

AAES	American Association of Endocrine Surgeons
ANCOVA	Analysis of Covariance
ATA	American Thyroid Association
AUC	Area Under the Curve
BAETS	British Association of Endocrine and Thyroid Surgeons
Ca	Calcium
CI	Confidence Interval
CND	Central Neck Dissection
ESE	European Society of Endocrinology
ESES	European Society of Endocrine Surgeons
GEE	Generalized Estimating Equations
hypoPTH	Hypoparathyroidism
IAES	International Association of Endocrine Surgeons
ICC	Intraclass Correlation Coefficient
ICG	Indocyanine Green
IONM	Intraoperative neuromonitoring
iPTH	Intact Parathyroid Hormone
IQR	Interquartile Range
LOS	Length of Stay
LR	Likelihood Ratio
NIRAF	Near-Infrared Autofluorescence
NRI	Net Reclassification Improvement
OR	Odds Ratio
PGRIS	Parathyroid Glands Remaining In Situ
POD1	Postoperative Day 1
PTC	Papillary Thyroid Carcinoma
PTH	Parathyroid Hormone
RCT	Randomized Controlled Trial
ROC	Receiver Operating Characteristic
RR	Relative Risk
RTM	Regression to the Mean
SQRTPA	Scandinavian Quality Register for Thyroid, Parathyroid and Adrenal Surgery
STROBE	Strengthening the Reporting of Observational Studies in Epidemiology
VIF	Variance Inflation Factor
ΔCa	Percent Change in Calcium
ΔPTH	Percent Change in Parathyroid Hormone
